# Effectiveness of sugar taxation policies in Asia and Africa: a systematic review

**DOI:** 10.3389/froh.2025.1520861

**Published:** 2025-04-09

**Authors:** Miyola Cia Fernandes, Praveen S. Jodalli, Deema Waleed Saeed, Ridhima Gaunkar, Sultan Almalki, Inderjit Gowdar, Aradhana Nagarsekar

**Affiliations:** ^1^ Centre for Health Policy, Asian Development Research Institute, Patna, Bihar, India; ^2^ Department of Public Health Dentistry, Manipal College of Dental Sciences Mangalore, Manipal Academy of Higher Education, Manipal, Karnataka, India; ^3^ Global Operations, Humble Smile Foundation India, Stockholm, Sweden; ^4^ Department of Public Health Dentistry, Goa Dental College and Hospital, Bambolim, India; ^5^ College of Dentistry, Prince Sattam Bin Abdulaziz University, Al-Kharj, Saudi Arabia; ^6^ Department of Prosthodontics, Goa Dental College and Hospital, Bambolim, India

**Keywords:** sugar tax, health policy, Asia, Africa, sugar, sugar sweetened beverages, SSB, health and wellbeing

## Abstract

**Background:**

The prevalence of major noncommunicable diseases (NCDs) such as cardiovascular disease, cancer, and diabetes is rising rapidly in Asia and Africa. One of the major modifiable risk factors for these diseases is the consumption of free sugars, commonly found in sugary drinks. To address this issue, some countries have implemented food taxes such as taxes on sugar-sweetened beverages as part of national public health policies to reduce its intake. The review aims to assess the effects of national taxation of sugar-sweetened beverages within the continents of Asia and Africa.

**Methods:**

Eight databases (MEDLINE (Ovid), Embase, PubMed, Cochrane, SCOPUS, Web of Science and ProQuest) were searched, and seven studies were included in this review. Only studies focused on the taxation of SSBs in Asia and Africa until 30 June 2023 and those that studied the impact of national sugar taxation among their population were included. Simulation or studies evaluating the estimation effect of taxes were excluded. All eligible records were assessed for the risk of bias using the NIH Quality Assessment Tool for Observational Cohort and Cross-Sectional Studies, and the certainty of the evidence was reviewed.

**Results:**

Seven studies included in this review investigated the impact of sugar tax policies in South Africa, India, Thailand, and Saudi Arabia. The interventions in these countries were implemented from 2017 to 2018 mainly for sugar-sweetened beverages. The studies provided evidence on changes in the volume of purchase, consumption, and sugar content of taxed items. Some evidence was found to suggest the positive impact of SSB taxes in reducing consumption of taxed items which ranged from 2.5% to 19% decrease. However, no study has reported on individual health outcomes.

**Conclusion:**

There is substantial evidence of a decrease in the consumption of taxed items, but there is uncertainty about the health impact of these outcomes. Future research should prioritize longitudinal studies assessing direct health impacts of SSB taxation policies. Additionally, generalizability of the results of such fiscal policies need to be investigated in lower economic settings and thus be of significance for uniform health policy reforms.

**Systematic Review Registration:** https://www.crd.york.ac.uk/PROSPERO/view/CRD42023427030, PROSPERO (CRD42023427030).

## 1 Background

The World Health Organisation (WHO) has identified cardiovascular diseases, respiratory diseases, cancers, and diabetes as the top noncommunicable diseases (NCDs) (1). Diet is a major modifiable risk factor for NCDs and contributes to overweight and obesity (2). According to the NCD Risk Factor Collaboration (NCD RisC), the overall global incidence of obesity has tripled since 1975, with approximately 671 million obese adults in 2016 according to worldwide pooled analysis (3).

The earlier misguided belief about the hazard of fats towards obesity has downplayed the role of other risk factors such as sugar. In contrast, the intake of free sugars and sugar-sweetened beverages (SSBs) have been found to be determinants of body weight (4).

Free sugars have been reported to be a common risk factor for type 2 diabetes (5, 6), cancer (6, 7), dental caries (8), high serum lipids (5) and obesity (5, 6).

Fiscal policies were suggested to be effective in promoting the nutritious dietary changes with the potential to improve healthy consumption at the population level (9).

For the prevention of dental caries, the dental community has often focused on downstream measures such as the application of fissure sealants and fluoride to treat the symptoms in high-risk individuals rather than a population-level reduction in sugar consumption.

According to the OECD/FAO 2019, in the next ten years, 98% of the additional demand for total world sugar consumption is expected to come from developing countries; in contrast, intake will continue to decline in developed countries due to increased consciousness about health and commercialisation of iso-glucose (a starch-based sweetener) in the sugar market (10). In developing and low-income countries, the intake of SSBs is on the rise, along with malnutrition (11).

The potential effects of interventions to reduce NCDs such as dental caries through the implementation of fiscal policies include increased purchase and consumption of healthy foods and decreased consumption of unhealthy foods, eventually decreasing dietary risk factors (12).

Taxes on sugar-sweetened products have been increasingly implemented by countries across the world (13, 14). This taxation on unhealthy foods and drinks leads to an increase in prices and reformulation led reduction in price, which eventually causes a decrease in sales, purchasing and consumption (15–22), as well as incentives for manufacturers to decrease production or reformulate unhealthy products. However, sugar taxation also requires equal support through incentivisation or cost subsidies to manufacturers and producers of healthy foods, advertisements, and health education, ultimately ensuring increased intake of a nutritious diet (22, 23). Additionally, it gives rise to revenue through excise collection, which can be invested in the health care system and boost health promotion activities (19, 24–28). SSB taxation can also result in unintended consequences from a fiscal policy environment leading to increased budget revenue (26) and undesired administrative government costs, which can elicit potential political influence (29).

A literature search on the impact of sugar taxation has shown that most of the studies (13, 18, 27, 28, 30–38) have focused mainly on high-income and middle-income countries, mainly within the American, European and Pacific regions (19). These studies have found post tax effects such as rise in SSB prices, reduced SSB consumption, reduced purchase of taxed SSBs and increased demand for alternative drinks (18, 19, 30, 34, 38). Economic evaluations of taxation of SSB taxes is found to be cost effective in six countries with savings from health care costs exceeding intervention costs (27, 33).

Systematic reviews involving evidence from simulation and modelling have reported that a higher taxation rate (15, 26, 39) in combination with other food subsidies (15, 40) would reduce the intake of sweetened items and prevent NCDs (27, 28); however, the impact would be inconsistent across socioeconomic groups (17, 41) and developing countries (42).

The continents of Asia and Africa comprise mainly of low and lower-middle income countries (43), where dietary patterns vary greatly as compared to westernised diet which is led by economic development and income stability (44). It is also important to note that in high-income countries, sugar consumption is socially patterned, with lower socioeconomic groups spending less money on food, leading to less unintended unhealthy food choices (12). However, in developing and low-income countries, the intake of SSBs is on the rise, along with malnutrition and obesity, as SSBs compensate for energy needs and decrease meal frequency (11) which is dependent on interaction of multiple factors such as social, economic, political, cultural, and biophysical (44). The results of effectiveness studies of sugar taxes could be diverse and unpredictable.

Thus a review of the available evidence on the effectiveness of sugar taxation policies in Asia and Africa is necessary to provide a picture of the current status. Thus, this study aims to provide up-to-date evidence of the effect of country-level sugar taxation policies enacted in Asia and Africa. This review will also provide an insight into forms of outcomes explored within this regions. Evidence of its effectiveness could prove to be instrumental in helping policymakers reform current health policies in these countries to reduce the risk of NCDs.

## 2 Methods

### 2.1 Study design

A systematic review was conducted to understand the impact of SSB taxes in Asia and Africa. The approach used here was exploratory information gathering and tabulation in a narrative synthesis format. This study was registered on PROSPERO (CRD42023427030).

### 2.2 Search strategy

A preliminary search was conducted via Google Scholar to identify keywords based on published abstracts and articles, which demonstrated the availability of very heterogeneous literature. This was followed by a systematic search in May 2023 using eight databases: MEDLINE (Ovid), Embase, PubMed, Cochrane, SCOPUS, Web of Science and ProQuest. Search strategies were enabled by Boolean operators (AND, OR, NOT), (e.g., sugar*), medical subject headings (MESH) and descriptive key terms where appropriate (Table 1). Eligible study references were followed up to identify other relevant records.

**Table 1:** Search terms for databases.

Database	Search terms	Inclusion
PubMed	[sugar(Title/Abstract)] AND [tax(Title/Abstract)]	Free full text
Science Direct	Title, abstract, keywords: sugar tax	
Wiley	“sugar” in Abstract AND “tax” in Abstract	Open Access Content Journals
SCOPUS	“sugar tax”	
Web of Science	sugar AND tax	
Embase	sugar AND tax	
ProQuest	sugar AND tax	
Cochrane	(impact):ti,ab,kw OR (effect):ti,ab,kw AND (sugar tax):ti,ab,kw	
	(sugar tax):ti,ab,kw	
	(sugar):ti,ab,kw AND (tax):ti,ab,kw	

### 2.3 Eligibility criteria

A review of SSBs in Asia and Africa was conducted to understand the impact of SSB taxes in these regions. All peer-reviewed literature published in English until 30 June 2023 that studied the impact of national sugar taxation among the population was eligible for review. There were no limitations placed on database exploration in terms of year of publication. The eligibility criteria are outlined in Table 2. Grey literature and non-peer reviewed literature were excluded to ensure higher prospect of credible, reliable, and accurate scientific information.

**Table 2:** Eligibility criteria.

Inclusion criteria	Exclusion criteria
National level taxes on SSBs in countries within Asia and Africa jurisdiction	Non SSB taxes and subsidies
Peer reviewed literature	State level taxes
Literature studying the direct changes that occurred as an impact of SSB taxes on the population and taxed products	SSB taxes in other countries
Literature published in English	Grey literature including literature from non-peer reviewed sources
Study design: All types of studies such as cross sectional, longitudinal, cohort studies, except estimation or projection effect studies.	Estimation or projection impact of SSB taxes
	Impact of other factors on SSB taxed products
	Literature in other languages

SSB, sugar sweetened beverages.

### 2.4 Data screening, selection, and extraction

**Figure 1:**
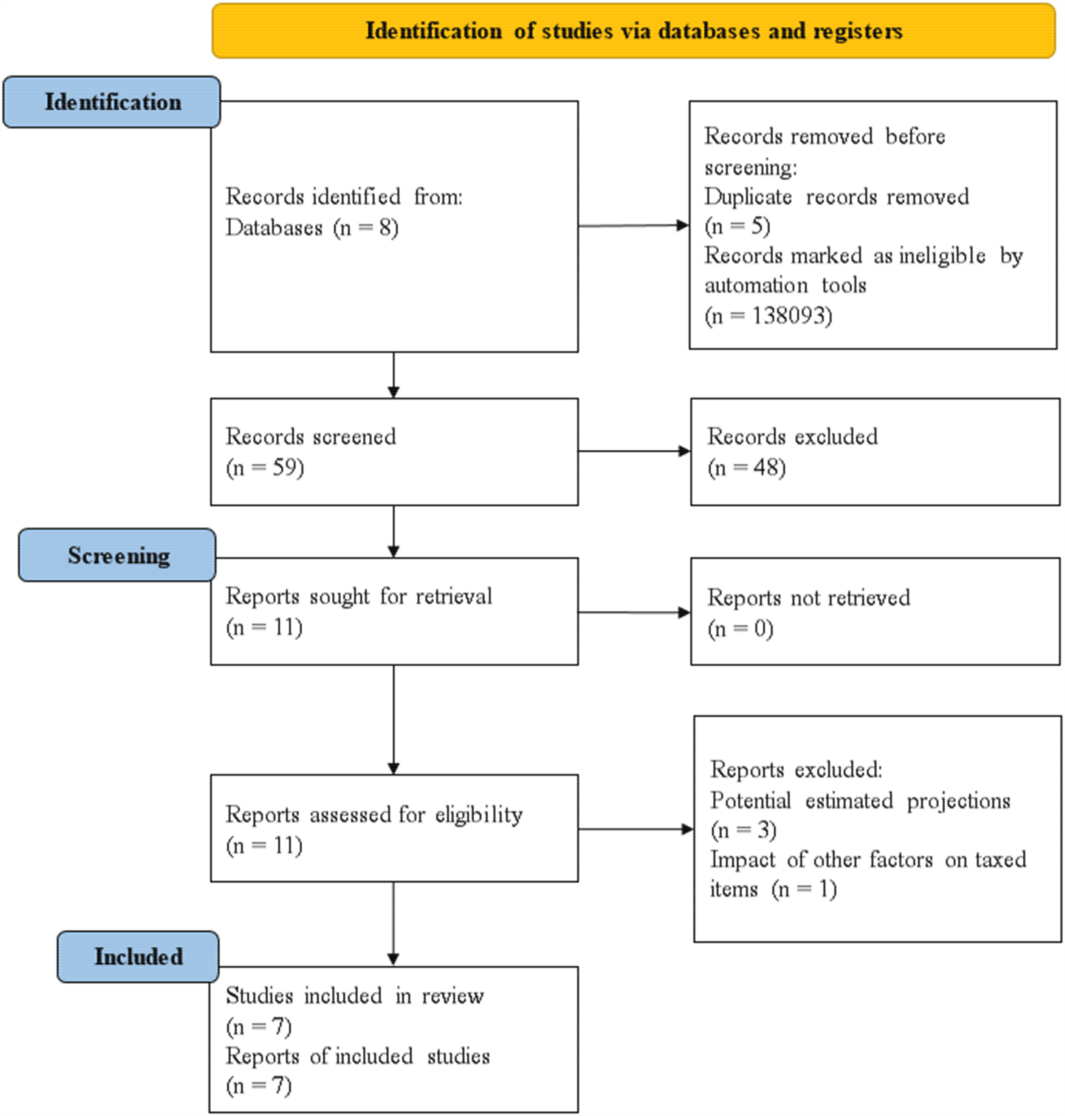
Study flow chart [PRISMA 2020 template – Page et al., (45)].

All findings were screened by the author (MCF) and reviewed by the author (RBG) to identify records that potentially met the eligibility criteria, followed by full-text screening, and the reasons for exclusions were recorded. For each study, information was extracted on taxation (such as type of tax, year of implementation and taxed products), study design (e.g., sample population, method of data collection, and statistical analysis), changes in SSBs (e.g., price, volume, and consumption) and population post implementation of the tax. The search strategy resulted in the inclusion of 7 studies (Figure 1).

The screening was conducted as follows: The authors screened the studies' titles, followed by screening of abstracts. If an abstract was not provided and the title appeared to be potentially relevant, the full text of the record was reviewed. Any disagreements were resolved by consensus and in consultation with a third review author, and all records that did not fit the inclusion criteria were excluded. The full texts of potentially relevant studies were retrieved for assessment and independently screened. At each stage, a record of the records retrieved and excluded was maintained. The PRISMA flowchart is presented in Figure 1 to display the selection of included studies.

All records were stored in reference management software (Endnote 2012). Author MCF independently extracted the data, which were reviewed by author RBG. The following data were extracted: publication type, country of study, funding source, type of study, participants, type of intervention, type of outcome measures, study methods, and results. If studies did not provide information on these criteria, the information was not extracted from these other sources. Qualitative data were not extracted.

### 2.5 Quality assessment

The NIH Quality Assessment Tool for Observational Cohort and Cross-Sectional Studies was used to assess risk of bias (46). This tool includes 14 dichotomous items such as the clarity of the research question or research objective; the definition, selection, composition, and participation of the study population; the definition and assessment of exposure and outcome variables; the measurement of exposures before outcome assessment; the study timeframe and follow-up; study analysis and power; and other factors (46). The studies were assigned a score of “1” if the criterion is present, for a total possible score of 14 (high quality). Author MCF independently evaluated the risk of bias of every included study and was then reviewed by author RBG.

We were not able to perform sensitivity analysis, robustness checks for missing data and meta-analysis as the reported research outcomes varied across all studies.

## 3 Results

Using the search strategy, a total of 7 studies were eligible and provided evidence of the effectiveness of sugar tax in Asia and Africa.

**Figure 2:**
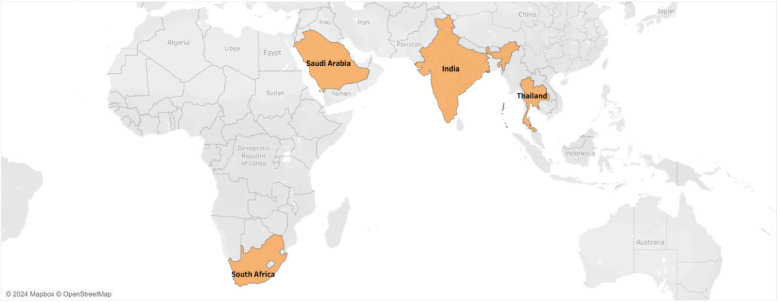
Overview of countries included in the studies.

Four countries, of which 3 belonged to Asia and 1 in Africa, had an SSB tax, the impact of which was evaluated in the eligible studies (see Figure 2).

The type of taxation policy varied among these countries (see Table 3). India had a GST on SSBs, while Saudi Arabia reported having an excise tax with a VAT, Thailand had an excise tax, and South Africa was found to have a levy.

**Table 3:** Overview of tax policies implemented in the included studies.

Sr. no	Author	Country studied	Type of taxation	Taxation rate	Products taxed	Tax implementation year
1	Bercholz et al., (47)	South Africa	Levy	2.1 c per gram of total sugar in excess of 4 g/100 ml (corresponds to 10% of retail price)	SSBs	2018
2	Law et al., (48)	India	GST	40%	Aerated drinks	
3	Megally and Al-Jawaldeh, (49)	Saudi Arabia	Excise tax	50%	SSB	2017
4	Jalloun and Qurban, (50)	Saudi Arabia	SSB tax + VAT	50% SSB tax + 5% VAT	SSB + energy drinks	2017
5	Stacey et al., (51)	South Africa	Levy	10%	SSB	2018
6	Essman et al., (52)	South Africa	Levy	2.1 cent for every gram of sugar above 4 g/ml threshold	SSB	2018
7	Phulkerd et al., (53)	Thailand	Excise tax		SSB	2017

SSB, sugar sweetened beverages; VAT, value added tax.

The rate of taxation varied in each of these countries (see Table 3), with the highest rate of 50% in Saudi Arabia. South Africa had a tiered taxation rate that varied across the level of sugar content in SSBs.

These countries implemented SSB taxation policies around the same timeframe of 2017–2018 (see Table 3).

These studies were funded by different global funding agencies whose authors have declared that they have no role in the methodology or publishing of results (see Table 5).

Four of the studies used interrupted time series data before and after intervention (48, 50, 52, 53) (Table 5), Megally et al. used time series data from 2010 to 2017 (49), and two studies (47, 51) used time series household data collected every month from 2014 to 2019.

The sample sizes and analytical methods applied in these studies differ widely and are reported in Table 5.

### 3.1 Outcomes

The interventions in all the studies involved the use of SSBs (Table 5). The outcomes were measured at different levels. Megally et al. (49) measured the outcome at the national level, Jalloun et al. (50), Phulkerd et al. (53) and Essman et al. (52) at the individual level, Stacey et al. (51) and Bercholz et al. (47) at the household level and Law et al. (48) at the state level.

### 3.2 Risk of bias assessment

The included studies were evaluated using the NIH Quality Assessment Tool for Observational Cohort and Cross-Sectional Studies. The overall quality rating for the internal validity varies for each study with scores ranging from 13 to 7 (Table 4). Two studies have high risk of bias due to unknown eligibility criteria and selection of participants. Most of the studies have adjusted for key confounding variables and there is need for further follow up to evaluate the further impact of exposure on outcomes. Additionally, the participants were not blinded due to the population level of interventions.

**Table 4:** Support for judgement in risk of bias assessment.

Criteria	Bercholz et al., (47)	Law et al., (48)	Megally and Al-Jawaldeh, (49)	Jalloun and Qurban, (50)	Stacey et al., (51)	Essman et al., (52)	Phulkerd et al., (53)	Summary
1. Was the research question or objective in this paper clearly stated?	Y	Y	Y	Y	Y	Y	Y	
2. Was the study population clearly specified and defined?	Y	Y	N	Y	Y	Y	Y	Megally et al. didn't provide any description about data collection
3. Was the participation rate of eligible persons at least 50%?	Y	NR	NR	Y	Y	Y	Y	Law et al, data wasn'tt adjusted by survey weight and thus not representative of urban India. Megally et al. didn't report about the eligibility.
4. Were all the subjects selected or recruited from the same or similar populations (including the same time period)? Were inclusion and exclusion criteria for being in the study prespecified and applied uniformly to all participants?	Y	Y	NR	Y	Y	Y	Y	Megally et al. didn't provide any description about the sample
5. Was a sample size justification, power description, or variance and effect estimates provided?	Y	N	N	Y	Y	N	Y	Law et al. it was difficult to understand when household entered and left the data panel. Megally et al. reported no sampling strategy. In Essman et al, all eligible households were invited to participate.
6. For the analyses in this paper, were the exposure(s) of interest measured prior to the outcome(s) being measured?	Y	Y	Y	Y	Y	Y	N	Phulkerd et al. evaluated both post tax study periods.
7. Was the timeframe sufficient so that one could reasonably expect to see an association between exposure and outcome if it existed?	Y	CD	Y	CD	Y	CD	Y	Three studies had only one Single exposure period was evaluated
8. For exposures that can vary in amount or level, did the study examine different levels of the exposure as related to the outcome (e.g., categories of exposure, or exposure measured as continuous variable)?	Y	Y	Y	Y	Y	Y	Y	
9. Were the exposure measures (independent variables) clearly defined, valid, reliable, and implemented consistently across all study participants?	Y	Y	Y	Y	Y	Y	Y	
10. Was the exposure(s) assessed more than once over time?	Y	N	Y	N	Y	N	Y	Three studies assessed only one post exposure time period
11. Were the outcome measures (dependent variables) clearly defined, valid, reliable, and implemented consistently across all study participants?	Y	Y	Y	Y	Y	Y	Y	
12. Were the outcome assessors blinded to the exposure status of participants?	N	N	N	N	N	N	N	Observational study lacking blinding of participants due to population level intervention
13. Was loss to follow-up after baseline 20% or less?	Y	NR	NR	Y	Y	Y	Y	Law et al. it was difficult to determine when household entered and left the data panel.
14. Were key potential confounding variables measured and adjusted statistically for their impact on the relationship between exposure(s) and outcome(s)?	Y	Y	N	Y	Y	Y	NR	Megally et al. and Phulkerd et al. didn't adjust for any confounding factors.
Overall score	13/14	8/14	7/14	11/14	13/14	10/12	11/14	

Y, yes; N, no; CD, cannot determine; NR, not reported; NA, not applicable.

### 3.3 Effect of interventions

The summary of findings in Table 5 presents an overview of the effects of the taxation of SSBs. Bercholz et al. (47) reported a change in price for taxed products of increase of 10%, which resulted in the discontinuation of taxable products, reformulation and a change in sugar content (Table 6). A smaller number of new taxes items were introduced post announcement of taxation policy as contrast to the discontinuation of 32% of taxed items (47).

**Table 5:** Summary of methodologies used in the included studies.

Sr. no.	Author	Type of study	Sample size	Data source	Data collection period	Secondary dataset	Statistical analysis	Funding
1	Bercholz et al., (47)	Cross-sectional longitudinal	3,000 households	Household purchase dataset from Europanel	January 2014 to March 2019	Mintel Global New Product database, nutrient dataset	Accounting decomposition, income level analysis, sensitivity analysis, descriptive analysis	Bloomberg Philanthropies, the South African Medical Research Council, the US NIH
2	Law et al., (48)	Cross-sectional longitudinal	48,490	State-level dataset from Kantar Worldpanel Division	January 2013 to June 2018		Interrupted time series analysis, sensitivity analysis	Wellcome Trust's Our Planet, Our Health Programme. In addition, the first author is funded via UK Medical Research Council Fellowship.
3	Megally and Al-Jawaldeh, (49)	Cross-sectional longitudinal			2010 to 2020	Secondary data by Global company intelligence	Regression analysis and Shapiro‒Wilk test	Eastern Mediterranean Regional Office of the World Health Organisation
4	Jalloun and Qurban, (50)	Cross-sectional	200	Online survey	April to May 2018		Logistic regressions	
5	Stacey et al., (51)	Longitudinal	113,653	Household purchase dataset from Kantar Europanel	Jan 2014 to March 2019	Nutrition panel data from multiple sources	Regression modelling	Bloomberg Philanthropies, the South African Medical Research Council, the US NIH
6	Essman et al., (52)	Cross sectional	Pretax 2,459 and post tax 2,489	Single day dietary recall through door to door household survey, nutrition facts panel data from grocery stores	Feb - March 2018, Feb - March 2019	Kantar world panel	Probit and linear modelling	Bloomberg Philanthropies, the University of Western Cape, the Population Research Infrastructure Program, the NIH training grant, the International Development Research Centre scholarships.
7	Phulkerd et al., (53)	Cohort	5,594	face to face interviews	may to dec 2018, June2019 to January 2020		t test	Sweet Enough Network

NIH, National Institute of Health.

**Table 6:** Overview of outcomes measured.

Sr. no.	Author	Change in price	Discontinued taxable products	Reformulation of taxable products to reduce sugar content	Change in sugar content	Changes in volume purchase	Change in consumption	Health related changes
1	Bercholz et al., (47)	10% rise in price	32% - post announcement, 21% - interim period	5.2% - interim, 17.6% (−0.3 g/capita/day) - post implementation, 43.6% (−1.4 g/capita/day) - post implementation	1.7 g/capita/day - interim, 3.1 g/capita/day - post implementation, 4.9 g/capita/day - overall decrease	38.8% (−0.7 g/capita/day) - interim; 26.3% (−0.8 g/capita/day) - post implementation		
2	Law et al., (48)					Beta = −0.008		
3	Megally and Al-Jawaldeh, (49)					Reduction of soft drink volume sales by 57.64% from 2010 to 2017		
4	Jalloun and Qurban, (50)						Soft drink consumption decreased by 19% among participants	Post taxation, not consuming soft drinks reduced risk of obesity by 32% as compared to 16% before taxation
5	Stacey et al., (51)					Volume of taxable purchase fell from 518.99 ml/capita per day to 492.16, while non-taxable beverage purchase increased from 283.45 ml/capita per day to 312.94		
6	Essman et al., (52)			Taxed beverages accounted for −22.2% reformulation of sugar intake		Volume intake of taxed beverages dropped by 117 ml/capita/day and increased by 340 ml/capita/day in untaxed beverages	Sugar intake decreased from 28.8 g/capita/day to 19.8 g/capita/day for taxed beverages and increased from 15.0 to 20.3 g/capita/day for untaxed beverages	
7	Phulkerd et al., (53)						Average daily SSB consumption decreased by 2.5%, with −2.8% in taxed SSB and −2.0% in untaxed SSB	

Additionally, share 17.1% share (47) and −22.2% sugar intake (52) of taxed beverages reformulated by reducing its sugar concentration post tax implementation (47). Bercholz et al. (47) also reported introduction of 14% rise in new non-taxed beverages post tax.

The change in the volume of sugar purchased differed, as Megally et al. (49) and Bercholz et al. (47) reported reductions of 57.64% and 26.3%, respectively, after the implementation of taxes. Stacey et al. (51) reported a minor decrease of 26.83 ml/capita per day in taxable beverages while non-taxable beverages rose by 29.49 ml/capita per day. Bercholz et al. (47) found switching accountable for 39.7% for reduction in sugar content of beverage purchases.

Essman et al. (52) showed a −117 ml/capita/day drop in volume intake of taxed beverages and 340 ml/capita/day increase in untaxed beverage volume.

The daily consumption of SSB decreased by 2.5% according to Phulkerd et al. (53), and soft drink consumption decreased by 19% according to Jalloun et al. (50), while Essman et al. (52) reported a decrease of 9 g/capita per day for taxed beverages after implementation. Sugar intake from untaxed beverages was seen to rise by 35.5% (52) and drop of 2% (53) post tax.

A −17.7% change was seen in taxed carbonated drinks, but sour milk/yogurt, freshly made herbal and iced teas showed an increase in Phulkerd et al. (53).

Jalloun et al. (50) reported that not consuming soft drinks after implementation reduced the risk of obesity by 32%.

### 3.4 Sensitivity analysis

Bercholz et al. (47) conducted sensitivity analysis to using energy levels of purchased data as sugar content was imputed only for 5% of available purchase data. Law et al. (48) analysed the percentage change of purchases by inclusion-exclusion of individual states and stratified analysis by income of states. Jalloun et al. (50) controlled for potential demographic confounding factors. Stacey et al. (51) compared regression-adjusted mean outcomes during both study periods. Essman et al. (52) conducted series of sensitivity analyses investigating impact of BMI on reporting intake; outcome dependent on missing LSM data; and beverages compensating for water shortage.

## 4 Discussion

### 4.1 Summary of the main results

Seven studies met the defined eligibility criteria for inclusion in our systematic review. We identified evidence on the effects of taxing sugar-added drinks on their volume, consumption, sugar content, and risk to health. However, Essman et al. (52) looked at the effects of other consumption-related outcomes, such as energy intake; Stacey et al. (51) also looked into expenditure-related outcomes, such as purchases of non-taxable beverages; and two studies (47, 52) analysed the impact of reformulation. Moreover, we found only one study (50) that examined the effects of taxing sugar-added beverages on the risk of health-related outcomes, such as obesity.

The findings from our review show that there is a substantial lack of evidence on the effects of taxing other sugar-added products, as we did not identify any study investigating this kind of intervention or its effects. According to the results of the included studies, the taxation of sugar-added beverages is effective for reducing consumption and purchase volume. The results indicated a varying reduction in consumption and purchase volume, but the certainty of the evidence is low because the sample was not a national distributive sample.

The effect on the mean consumption of untaxed sugar-added drinks increased in 2 studies (51, 52) and decreased by a small margin in the study by Phulkerd et al. (53). Thus, the certainty of the increase in consumption of non taxed items due to the substitution effect is low, and the difference in the consumption of taxed and untaxed sugar-added foods compared to untaxed sugar-added foods in Phulkerd et al. (53) is small (0.8%).

There is no evidence on the impact of the taxation of sugar-added drinks on reducing expenditures.

The study results could not be pooled or combined with interventions to perform a meta-analysis.

### 4.2 Implications for policy and practice

Implementation of SSB taxation can also lead to substitution effect by causing a shift in uptake of sugar containing non taxed items as well as other dietary products. But these changes require a long-term longitudinal evaluation to understand its outcomes.

Sugar taxation also has its unintended implication in the form of public resistance or increase in purchase of taxed items from untaxed regions. A potential economic and inequity impact may arise due to increased tax burden on low social economic groups who have reported higher intake of SSBs to compensate for energy needs.

Although lower income countries will contribute financially from implementation of sugar taxation, additional support through government incentives to reduce cost of healthy food items are necessary to make the taxation policy less regressive towards lower income populations. Care must be taken to understand the heterogeneity of health taxation policies across various population sub groups.

Health tax such as sugar taxation require to be supplemented with equal amount of health awareness programs highlighting the ill-effects of unhealthy products along with introduction of healthier dietary products thereby providing an all-round drive to tackle non-communicable diseases and reduced health costs.

Imposing a universal sugar tax rate of 20% might not be the most prudent choice without supporting healthy sustainable incentives, as a large portion of the population belongs to lower economic groups, where socio-economic, cultural, commercial, and religious determinants of health place a significant burden on the quality of lives in this demographic.

### 4.3 Overall completeness and applicability of evidence

The objectives of this review are sufficiently addressed. The existing evidence in this review was derived from seven studies across four countries belonging to the lower-middle (India), upper-middle (South Africa, Thailand) and higher (Saudi Arabia) income classifications of countries (43); thus, the evidence is limited with respect to comparability to poorer nations.

The available evidence needs to be improved, as the results might be biased due to the presence of other interventions and taxation policies as well as the misclassification of taxed items. Accurate reporting and measurement of consumption data is challenging and might produce recall bias. Comparability of the results from the included studies is challenging due to vast distinction in taxation type, rates as well as outcome measures. Some studies might have looked at common outcome measures, but the unit of measure differs, in addition to the variation in data collection methodology.

For the reasons outlined, further evidence is required to improve its applicability.

### 4.4 Agreements and disagreements with other studies or reviews

There have been no previously conducted systematic reviews on the effects of taxing unprocessed sugar or sugar-added foods in Asia and Africa. However, systematic reviews in other regions have been conducted (13, 18, 19, 30–37). Systematic review involving a mix of high and middle-income countries concluded that high SSB tax rates along with other preventive interventions are needed to induce positive health outcomes (18, 30, 32). Another review based on high-income countries found that taxes framed around health promotion have higher public, media and policy communities support, however industry interests have caused abolishment of health taxes (19). Meta analysis of global sugar taxation policies found a drop of 15% in mean sales of taxed items and −1.59 price elasticity demand (13).

However, the evidence base in the mentioned reviews has low applicability to the objective regions of this review. Food consumption patterns are changing globally with increased as liberalisation along with conflicts in Asia and Africa have led to increased food prices, marketing of unhealthy products and reduced diet quality. As with the population-level interventions in the existing reviews, the policies, along with the methodological approaches and population settings, are completely different from those in our review and thus cannot be compared.

There was no clinical individual-level significance found in this review. However, taxing sugar-added drinks is meaningful at the population level and thus of significance for health policy reforms. The results of this review were derived from four countries, but the generalizability of the results to populations in lower economic settings is uncertain. Additionally, evidence of the effect of taxing sugar-added beverages on health outcomes is very low, and therefore, caution is required in its application to improve health outcomes.

These findings demonstrate the need for further research to investigate the effectiveness of sugar taxes on expenditures and health-related outcomes.

In summary, there is sufficient evidence that the taxation of SSBs is effective in reducing their consumption.

### 4.5 Quality of the evidence

For the taxation of sugar-added beverages, the certainty of evidence of consumption and purchase volume is uncertain. There is no evidence on the impact on expenditure and health outcomes. Therefore, the real effect may differ substantially from the expected outcomes.

Two studies were downgraded due to non-reporting of participation rate. Three studies were also downgraded due to the need for further follow up as the current time frame of the study is insufficient to determine an association between exposure and outcome. Another 3 studies were downgraded due to single follow-up post exposure. Two studies were downgraded due to lack of reporting of loss to follow up. Two studies did not adjust or did not report about potential confounding factors impacting the relationship between outcome and exposure.

### 4.6 Potential biases in the review process

The risk of bias in the review process was potentially low, as all eligible studies were included in this review. The search strategy, database searches, extracted data, screened titles, abstracts and full texts were reviewed by a second author.

## 5 Conclusion

Although evidence of a reduction in the consumption and purchase volume of sugar-containing beverages after taxation has been reported, the effectiveness of taxing SSBs for reducing adverse health outcomes is very limited. No studies have investigated the impact of taxing sugar-added drinks on health-related outcomes that could be used to derive great implications for practice.

Further studies providing greater evidence are required to assess the effectiveness of taxing food items for reducing adverse health outcomes. Most of these taxes have been implemented recently and thus provide great potential to investigate their impact for further studies. Future research is particularly needed in all countries with sugar taxation to assess the wider effects of taxes on dietary items, with special attention given to considering health impacts as relevant outcome domains.

## Data Availability

The original contributions presented in the study are included in the article/Supplementary Material, further inquiries can be directed to the corresponding authors.

## References

[1] WHO. Noncommunicable Diseases. World Health Organisation (2018). Available at: https://www.who.int/news-room/fact-sheets/detail/noncommunicable-diseases (Accessed March 24, 2024).

[2] WHO. Reducing free sugars intake in adults to reduce the risk of noncommunicable diseases. World Health Organisation (2019). Available at: https://www.who.int/elena/titles/free-sugars-adults-ncds/en/ (Accessed March 24 2024).

[3] NCD Risk Factor Collaboration. Worldwide trends in body-mass index, underweight, overweight, and obesity from 1975 to 2016: a pooled analysis of 2416 population-based measurement studies in 128.9 million children, adolescents, and adults. Lancet. (2017) 390(10113):2627–42. 10.1016/S0140-6736(17)32129-3 29029897 PMC5735219

[4] Te Morenga L Mallard S Mann J. Dietary sugars and body weight: systematic review and meta-analyses of randomised controlled trials and cohort studies. Br Med J. (2012) 346(jan15 3):e7492–2. 10.1136/bmj.e7492 23321486

[5] Hauner H Bechthold A Boeing H Brönstrup A Buyken A Leschik-Bonnet E Evidence-based guideline of the German nutrition society: carbohydrate intake and prevention of nutrition-related diseases. Ann Nutr Metab. (2012) 60(s1):1–58. 10.1159/000335326 22286913

[6] Malik VS Hu FB. The role of sugar-sweetened beverages in the global epidemics of obesity and chronic diseases. Nat Rev Endocrinol. (2022) 18(4):205–18. 10.1038/s41574-021-00627-6 35064240 PMC8778490

[7] Makarem N Bandera EV Nicholson JM Parekh N. Consumption of sugars, sugary foods, and sugary beverages in relation to cancer risk: a systematic review of longitudinal studies. Annu Rev Nutr. (2018) 38(1):17–39. 10.1146/annurev-nutr-082117-051805 29801420

[8] Sheiham A James WPT. Diet & dental caries: the pivotal role of free sugars reemphasised. J Dent Res. (2015) 94(10):1341–7. 10.1177/0022034515590377 26261186

[9] Thow AM Downs S Jan S. A systematic review of the effectiveness of food taxes and subsidies to improve diets: understanding the recent evidence. Nutr Rev. (2014) 72(9):551–65. 10.1111/nure.12123 25091552

[10] OECD/FAO. OECD-FAO Agricultural Outlook 2019–2028. Rome: OECD Publishing, Paris/Food and Agriculture Organisation of the United Nations (2019). https://www.oecd-ilibrary.org/agriculture-and-food/oecd-fao-agricultural-outlook-2019-2028_agr_outlook-2019-en (accessed March 20 2024).

[11] Imamura F Micha R Khatibzadeh S Fahimi S Shi P Powles J Dietary quality among men and women in 187 countries in 1990 and 2010: a systematic assessment. Lancet Glob Health. (2015) 3(3):e132–42. 10.1016/S2214-109X(14)70381-X 25701991 PMC4342410

[12] Pechey R Monsivais P. Socioeconomic inequalities in the healthiness of food choices: exploring the contributions of food expenditures. Prev Med. (2016) 88:203–9. 10.1016/j.ypmed.2016.04.012 27095324 PMC4910945

[13] Andreyeva T Marple K Marinello S Moore TE Powell LM. Outcomes following taxation of sugar-sweetened beverages: a systematic review and meta-analysis. JAMA Network Open. (2022) 5(6):e2215276. 10.1001/jamanetworkopen.2022.15276 35648398 PMC9161017

[14] Obesity Evidence Hub. Countries that have taxes on sugar-sweetened beverages (SSBs). Available at: https://www.obesityevidencehub.org.au/collections/prevention/countries-that-have-implemented-taxes-on-sugar-sweetened-beverages-ssbs (Accessed January 21 2025).

[15] Maniadakis N Kapaki V Damianidi L Kourlaba G. A systematic review of the effectiveness of taxes on nonalcoholic beverages and high-in-fat foods as a means to prevent obesity trends. Clinicoecon Outcomes Res. (2013) 5:519–43. 10.2147/CEOR.S49659 24187507 PMC3810203

[16] Niebylski ML Redburn KA Duhaney T Campbell NR. Healthy food subsidies and unhealthy food taxation: a systematic review of the evidence. Nutrition. (2015) 31(6):787–95. 10.1016/j.nut.2014.12.010 25933484

[17] Backholer K Sarink D Beauchamp A Keating C Loh V Ball K The impact of a tax on sugar-sweetened beverages according to socioeconomic position: a systematic review of the evidence. Public Health Nutr. (2016) 19(17):3070–84. 10.1017/S136898001600104X 27182835 PMC10270974

[18] Nakhimovsky SS Feigl AB Avila C O’Sullivan G Macgregor-Skinner E Spranca M. Taxes on sugar-sweetened beverages to reduce overweight and obesity in middle-income countries: a systematic review. PLoS One. (2016) 11(9):e0163358. 10.1371/journal.pone.0163358 27669014 PMC5036809

[19] Wright A Smith KE Hellowell M. Policy lessons from health taxes: a systematic review of empirical studies. BMC Public Health. (2017) 17:1–4. 10.1186/s12889-016-3954-4 28629470 PMC5477308

[20] Redondo M Hernández-Aguado I Lumbreras B. The impact of the tax on sweetened beverages: a systematic review. Am J Clin Nutr. (2018) 108(3):548–63. 10.1093/ajcn/nqy135 30535085

[21] Teng AM Jones AC Mizdrak A Signal L Genç M Wilson N. Impact of sugar-sweetened beverage taxes on purchases and dietary intake: systematic review and meta-analysis. Obes Rev. (2019) 20(9):1187–204. 10.1111/obr.12868 31218808 PMC9285619

[22] Urwannachotima N Hanvoravongchai P Ansah JP Prasertsom P Koh VRY. Impact of sugar-sweetened beverage tax on dental caries: a simulation analysis. BMC Oral Health. (2020) 20(1):1–12. 10.1186/s12903-020-1061-5 PMC7079374 32183817

[23] Ells LJ Roberts K Mcgowan VJ Machaira T. Sugar Reduction: The Evidence for Action. Annexe 2: A Mixed Method Review of Behaviour Changes Resulting from Experimental Studies That Examine the Effect of Fiscal Measures Targeted at High Sugar Food and non-alcoholic drink. London: Public Health England (2015). p. 87.

[24] Timpson H Lavin R Hughes L. Exploring the Acceptability of a tax on Sugar-sweetened beverages: Insight Work. Liverpool: Centre for Public Health, Liverpool John Moores University (2013).

[25] Eykelenboom M van Stralen MM Olthof MR Schoonmade LJ Steenhuis IHM Renders CM Political and public acceptability of a sugar-sweetened beverages tax: a mixed-method systematic review and meta-analysis. Int J Behav Nutr Phys Act. (2019) 16(1):78. 10.1186/s12966-019-0843-0 31484538 PMC6727579

[26] Widarjono A Afin R Kusnadi G Firdaus MZ Herlinda O. Taxing sugar sweetened beverages in Indonesia: projections of demand change and fiscal revenue. PLoS One. (2023) 18(12):e0293913. 10.1371/journal.pone.0293913 38157352 PMC10756547

[27] Salgado Hernandez JC Ng SW Stearns SC Trogdon JG. Cost-benefit analysis of alternative tax policies on sugar-sweetened beverages in Mexico. PLoS One. (2023) 18(10):e0292276. 10.1371/journal.pone.0292276 37788248 PMC10547152

[28] Lee MM Barrett JL Kenney EL Gouck J Whetstone LM McCulloch SM A sugar-sweetened beverage excise tax in California: projected benefits for population obesity and health equity. Am J Prev Med. (2024) 66(1):94–103. 10.1016/j.amepre.2023.08.004 37553037 PMC10840962

[29] Alvarado M Adams J Penney T Murphy MM Abdool Karim S Egan N A systematic scoping review evaluating sugar-sweetened beverage taxation from a systems perspective. Nat Food. (2023) 4(11):986–95. 10.1038/s43016-023-00856-0 37857862 PMC10661741

[30] Cabrera Escobar MA Veerman JL Tollman SM Bertram MY Hofman KJ. Evidence that a tax on sugar sweetened beverages reduces the obesity rate: a meta-analysis. BMC Public Health. (2013) 13:1–0. 10.1186/1471-2458-13-1072 24225016 PMC3840583

[31] Miracolo A Sophiea M Mills M Kanavos P. Sin taxes and their effect on consumption, revenue generation and health improvement: a systematic literature review in Latin America. Health Policy Plan. (2021) 36(5):790–810. 10.1093/heapol/czaa168 33885782 PMC8173601

[32] Teng A Snowdon W Tin ST Genç M Na'ati E Puloka V Progress in the Pacific on sugar-sweetened beverage taxes: a systematic review of policy changes from 2000 to 2019. Aust N Z J Public Health. (2021) 45(4):376–84. 10.1111/1753-6405.13123 34097355

[33] Liu S Veugelers PJ Liu C Ohinmaa A. The cost effectiveness of taxation of sugary foods and beverages: a systematic review of economic evaluations. Appl Health Econ Health Policy. (2022) 20(2):185–98. 10.1007/s40258-021-00685-x 34608610

[34] Pfinder M Heise TL Boon MH Pega F Fenton C Griebler U Taxation of unprocessed sugar or sugar-added foods for reducing their consumption and preventing obesity or other adverse health outcomes. Cochrane Database Syst Rev. (2020) 4(4):CD012333. 10.1002/14651858.CD012333.pub2 32270494 PMC7141932

[35] WHO. Fiscal policies for diet and prevention of noncommunicable diseases: technical meeting report, 5-6 May 2015, Geneva, Switzerland. World Health Organisation (2016).

[36] WHO. Healthy Islands: Best Practices in Health Promotion in the Pacific. Manila: World Health Organisation (2017).

[37] McDonald A. Sugar-sweetened Beverage tax in Pacific Island Countries and Territories: A Discussion Paper. Noumea: Secretariat of the Pacific Community (2015).

[38] Salgado Hernández JC Ng SW Colchero MA. Changes in sugar-sweetened beverage purchases across the price distribution after the implementation of a tax in Mexico: a before-and-after analysis. BMC Public Health. (2023) 23(1):265. 10.1186/s12889-023-15041-y 36750794 PMC9906831

[39] Itria A Borges SS Rinaldi AE Nucci LB Enes CC. Taxing sugar-sweetened beverages as a policy to reduce overweight and obesity in countries of different income classifications: a systematic review. Public Health Nutr. (2021) 24(16):5550–60. 10.1017/S1368980021002901 34218837 PMC10195460

[40] Afshin A Penalvo JL Del Gobbo L Silva J Michaelson M O'Flaherty M The prospective impact of food pricing on improving dietary consumption: a systematic review and meta-analysis. PLoS One. (2017) 12(3):e0172277. 10.1371/journal.pone.0172277 28249003 PMC5332034

[41] Jain V Crosby L Baker P Chalkidou K. Distributional equity as a consideration in economic and modelling evaluations of health taxes: a systematic review. Health Policy. (2020) 124(9):919–31. 10.1016/j.healthpol.2020.05.022 32718790

[42] Shakiba M Iranparvar P Jadidfard MP. The impact of sugar-sweetened beverages tax on oral health-related outcomes: a systematic review of the current evidence. Evid Based Dent. (2022):1–6. 10.1038/s41432-022-0830-1 36477678

[43] The World Bank. World Bank Country and Lending Groups. The World Bank (2024). Available at: https://datahelpdesk.worldbank.org/knowledgebase/articles/906519-world-bank-country-and-lending-groups (Accessed March 20 2024).

[44] da Costa GG da Conceição Nepomuceno G da Silva Pereira A Simões BF. Worldwide dietary patterns and their association with socioeconomic data: an ecological exploratory study. Global Health. (2022) 18(1):31. 10.1186/s12992-022-00820-w 35279165 PMC8917745

[45] Page MJ McKenzie JE Bossuyt PM Boutron I Hoffmann TC Mulrow CD The PRISMA 2020 statement: an updated guideline for reporting systematic reviews. Br Med J. (2021) 372:n71. 10.1136/bmj.n71 33782057 PMC8005924

[46] National Heart, Lung and BI (NHLBI). Study quality assessment tools (2018). Available at: https://internet-prod.nhlbi.nih.gov/health-topics/study-quality-assessment-tools (Assessed January 20, 2025).

[47] Bercholz M Ng SW Stacey N Swart EC. Decomposing consumer and producer effects on sugar from beverage purchases after a sugar-based tax on beverages in South Africa. Econ Hum Biol. (2022) 46:101136. 10.1016/j.ehb.2022.101136 35358759 PMC9288974

[48] Law C Brown KA Green R Venkateshmurthy NS Mohan S Scheelbeek PF Changes in take-home aerated soft drink purchases in urban India after the implementation of goods and services tax (GST): an interrupted time series analysis. SSM Popul Health. (2021) 14:100794. 10.1016/j.ssmph.2021.100794 33997244 PMC8102159

[49] Megally R Al-Jawaldeh A. Impact of sin taxes on consumption volumes of sweetened beverages and soft drinks in Saudi Arabia. F1000Res. (2020) 9:1117. 10.12688/f1000research.25853.1 33510893 PMC7809884

[50] Jalloun RA Qurban MA. The impact of taxes on soft drinks on adult consumption and weight outcomes in medina, Saudi Arabia. Hum Nutr Metab. (2022) 27:200139. 10.1016/j.hnm.2022.200139

[51] Stacey N Edoka I Hofman K Swart EC Popkin B Ng SW. Changes in beverage purchases following the announcement and implementation of South Africa’s health promotion levy: an observational study. Lancet Planet Health. (2021) 5(4):e200–8. 10.1016/S2542-5196(20)30304-1 33838735 PMC8071067

[52] Essman M Taillie LS Frank T Ng SW Popkin BM Swart EC. Taxed and untaxed beverage intake by South African young adults after a national sugar-sweetened beverage tax: a before-and-after study. PLoS Med. (2021) 18(5):e1003574. 10.1371/journal.pmed.1003574 34032809 PMC8148332

[53] Phulkerd S Thongcharoenchupong N Chamratrithirong A Soottipong Gray R Prasertsom P. Changes in population-level consumption of taxed and nontaxed sugar-sweetened beverages (SSB) after implementation of SSB excise tax in Thailand: a prospective cohort study. Nutrients. (2020) 12(11):3294. 10.3390/nu12113294 33121147 PMC7692763

